# Investigating Information-Seeking Behavior of Faculty Members Based on Wilson’s Model: Case Study of PNU University, Mazandaran, Iran

**DOI:** 10.5539/gjhs.v8n9p26

**Published:** 2015-10-16

**Authors:** Fereydoon Azadeh, Shahrzad Ghasemi

**Affiliations:** 1 Assistant Professor, Faculty of Allied Medical Sciences, Tehran University of Medical Sciences, Tehran, Iran; 2 Library and Information Sciences, Tonekabon Branch, Islamic Azad University, Tonekabon, Iran

**Keywords:** information seeking behavior, faculty members, internet-based resources, Wilson’s model

## Abstract

The present research aims to study information seeking behavior of faculty Members of Payame Noor University (PNU) in Mazandaran province of Iran by using Wilson’s model of information seeking behavior. This is a survey study. Participants were 97 of PNU faculty Members in Mazandaran province. An information-seeking behavior inventory was employed to gather information and research data, which had 24 items based on 5-point likert scale. Collected data were analyzed in SPSS software. Results showed that the most important goal of faculty members was *publishing a scientific paper*, and their least important goal was *updating technical information*. Also we found that they mostly use internet-based resources to meet their information needs. Accordingly, 57.7% of them find information resources via online search engines (e.g. Google, Yahoo). Also we concluded that there was a significant relationship between *English language proficiency*, *academic rank*, and *work experience* of them and their information- seeking behavior.

## 1. Introduction

Humans may face several ambiguous questions and phenomena every day. They require information to answer these questions and discover these phenomena. Indeed, Information seeking is required to obtain data, and it is a process during which the individual moves from unbeknown towards knowledge and awareness. But in the present era, we encounter the huge amount of information i.e., information explosion. Therefore, it should be noted that those individuals who can use these information, will have a limited ability to examine all of them. In fact, they would rather have appropriate and accurate information to a high amount of information. In other words, not only accessing information but also accessing information which are tailored to the information needs is of great importance. The behavior which an individual shows on track to get the answers to his questions indicates his information seeking behavior. Up to now, several researches have been conducted on the individuals’ information seeking behaviors and patterns. These researches illustrate that researchers have applied different methods for gaining information under the effect of different factors. Therefore, information seeking behavior of the researchers can be guided to specific and targeted routes through knowing and controlling these factors. In fact, information seeking patterns provide the relationships, theoretical cases, and processes which are related to identifying and answering information needs.

Nowadays, internet is used by millions of users as one of the main channels of accessing information. Students are interested in this media, because it provides them various facilities both in terms of accessing huge amount of information and, communications and contextualizing sharing information. This network has such an effect on research and training processes that nowadays accessing internet in universities cannot be ignored, and annually, a noticeable part of universities budgets is allocated to create infrastructures and accessing internet sources of information including hardware and software requirements, human sources, and so on (Davarpanah, 2002).

Nowadays wherein scientific communities benefit from extensive communication facilities, it is expected that the researchers and the scholars update their knowledge, and use the available printed and electronic information. The use of internet and its various features is one if the ways by which easy and quick access to scientific information is achieved. Information resources are any printed or electronic sources containing available information for responding (Nokarizi, & Davarpanah, 2006). Access to information is not the only criterion; accessing to information tailored to information needs is the matter as well. The individual’s behavior towards getting the answers of the questions and discovering new knowledge indicates his information seeking behavior. According to Wilson (2000), information seeking behavior is the micro-level of behavior employed by the researcher in interacting with all kinds of systems. It consists of all the interactions with the systems, whether at the level of human computer interaction (e.g., the use of the mouse and clicks on the mouse) or at the intellectual level (e.g., determining the criteria for deciding which of the two books selected from adjacent places on a library shelf is most useful).

Researchers apply different methods to gain information influenced by different factors, and consequently, show a different information seeking behavior. So, the researchers’ information seeking behavior can be led to specified and purposeful directions by true recognition and control of these factors. According to Marchionini (1995), the characteristics of information seeking is solving the problem. It includes a. recognizing and accepting an information problem b. defining the problem c. choosing a search system d. formulating a query e. executing search e. examining results f. extracting information g. reflecting, iterating or stopping (Choo et al, 2000). Since information technology has profoundly affected both research and researchers in Iran, and internet plays an important role in Iranian people information seeking behaviors, especially its faculty members. This research aims to examine the effect of using internet on the information seeking behavior of faculty members of PNU in Mazandaran province of Iran by using the Wilson’s model.

## 2. Materials and Methods

### 2.1 Information Seeking and Information Seeking Behavior

By increasing the rate and number of using computers in houses, workplaces, and during leisure times, and by expansion of digital data, dissemination of information in various forms in internet and World Wide Web, the search of information by users search will also increase (Cooke, 2001). Although information revolution is not an issue that has begun recently, the perception of information seeking behavior is a vital issue in this age. In future designs, extraordinary efforts should be made to establish intelligent systems which examine users. Finally, new technology is not responsible for success or failure in this, but it requires awareness of human needs and his behavior to access the information. Information seeking is vital and necessary for human. People use information seeking to meet their information needs. According to Marchionini (1995), the process of information seeking is originated from human life. In the pre-digital era, humans sought information in various fields such as finding the most suitable place to live, the best way to haunt, the ways of patient care, and so on. As the technology develops, other media are created and data are transferred fast. Marchionini (1995) believes that the information seeking is a way to solve the problem, and it depends on both information searcher and system. He also defines information seeking as a process in which the human attempts purposefully to change the status of knowledge. As we know, people use information to solve their problems, do a job, or increase the level of perception. Therefore, more recognition of information seeking as a social behavior help us have more information developments, and design better information systems.

After the World War II, the conducted researches on information seeking behavior led to make an apparent change in seeking behavior. In those years, some comprehensive methods were developed for the evaluation of needs and the use of information. This issue was a positive step in further researches, and provided an attractive space for designing software and hardware interface. New aspects of the researches changed traditional models of design and caused users to be in the center of attention instead of system. Information seeking behavior is human complex patterns of behavior as well as mutual interactions during searching any type of information. This conception is related to the users’ field study. Lancaster remarks that information retrieval is a process of searching among a set of information resources which aims to determine a specific collection of information resources in the field of a certain subject (Gazni, 2002).

Krikelas (1983) believes that information seeking behavior is the individual’s attempt for message recognition due to a perceived need. Those individuals whose information needs are determined show some behaviors for using information resources. Wilson uses four terms of “information behavior”, “information seeking behavior”, “information searching behavior”, and “information use behavior”, and distinguishes them from each other. According to him, *information behavior* means the totality of human behavior to sources and channels of information, including active and passive information seeking, and information use. Therefore, it includes face-to-face communication with others and passive reception of information such as watching TV without any intention to do any certain act on the given information. *Information seeking behavior* is purposeful searching for information to satisfy a certain goal. In the course of searching, the individual may interact with manual information system (e.g., newspaper or library) or internet-based systems (e.g., World Wide Web).*Information searching behavior* is a micro-level of behavior which the searcher employs to interact with information systems. It includes all the interactions with system at the level of human interaction with internet (such as using mouse and clicks on links) or at the intellectual level (such as adopting Boolean search strategy or determining some criteria to make decision on which one of two books selected from adjacent places of a library is most readable) which involves mental acts such as judging the relevance of retrieved data or information. Information use behavior is consisted of mental and physical acts based on the information found into the person’s knowledge base. Marking some sections in a text to show their importance is an example of physical act, and comparison of new information with existing knowledge is an example of mental act (Wilson, 2000).

### 2.2 Information Searching Behavior and Information Searchers

Awareness of this fact that information searching not simply means searching information, but it focuses on the use of interesting, conceptual, edited, and organized information is of high importance. According to the researches undertaken in this field, information needs, choosing related information, searching accurate information, and the use of information are considered as the most important components of information searching, and the main goal of information searching is to access accurate information. Every individual has his own point of view affected by different factors such as available information resources, personal factors, and career, social, political, and economic fields which can lead him to gain more useful information (Makri et al., 2008).

According to Ellis et al. (1993), there are eight key stages within the information seeking process including stating, chaining, browsing, differentiating, monitoring, extracting, verifying, and ending. He believes that every searcher has different level of accuracy and attention in each of the above stage based on his experience, information need, and expectancy level. Finally, this is the searcher that select and collect information based on his need (Meho & Tibo, 2003). Ellis proposed a model for searchers’ information seeking behavior. He has stated that this model can be implemented in closed environment such as databases wherein special instruments like controlled vocabulary is used.

Saracevic (2002) believes that these models present the interaction between system and user. He proposed a model which has pointed out well that how hidden factors such as cognitive changes affect visible changes such as re-formulating the question. These factors can cause us to show both active and dynamic information seeking behavior. Most of the times, we show the first one. For example, when we are watching TV or listening to music, we deal with active information seeking. Also, when we are searching for further information in search engines or databases, we unexpectedly access the related information and other useful information; we show an active information seeking behavior. The information which is gained through this way is likely to be a source for novel and extensive researches. In contrast, dynamic information seeking derives from movement towards the pre-determined goals, re-implementation of search orders, limiting the search scope, and updating our mental knowledge (Bronstein, 2007).

Some researchers such as Ellis et al. (1993) and Choo et al. (2000) have considered the process of information seeking as the process of information reviewing. The researches show that information searchers are divided into three main groups: beginner searchers, specialist searchers, and top socialist searchers or searchers who are interested in improving search protective mechanisms. It is clear that the information seeking behavior cannot be considered similar among these three groups who have different level of knowledge and experience of searching, because each of them searches according to his certain needs and also the level of his skills, knowledge, and experience. Due to this fact, they will employ different information seeking behavior. These groups differ from each other in terms of their information needs, and also searching for their required data or information. From another point of view, since real searchers are always searching for the best and the most valuable information, and preparing and organizing these sources make libraries and informing centers to pay higher costs, these centers make individuals to pay money to access these information resources, so, costs of accessibility can be considered as one of the most effective factors on users’ information seeking behavior (Eslami, & Keshavarz, 2006).

Sometimes, considering some overall costs for accessing information can make change in searching and searcher’s information seeking behavior which leads both of them to be conducted in another way. Specialist, non-specialist, organizational, and technical searchers are not as same as beginners and novices. Because of this, the costs paid for accessing information cannot be considered as an effective and intervening factor for the first group who usually seek information while are supported by an efficient organization, and have more special and considerable information needs rather than the beginner and novice searchers, but this factor can be effective for the searchers of second group including beginner and novice users which lead them to use free information. Of course, if the tendency towards the use of free information is along with searcher’s information literacy such as lacking sufficient abilities and skills in identifying and evaluating valuable information, the searchers’ informing will include some invaluable information which directly affect their research results. Because of this, the factor of cost can be considered as an effective factor in searchers’ information seeking behavior (Eslami, & Keshavarz, 2006).

### 2.3 Information Seeking Skills

4 kinds of Information seeking behavior skills are: retrieving, evaluating, organizing, and interchanging information.

- Information retrieval skills, including the recognition of information resources related to having skill in searching strategies, and the ability to use indexes and abstracts
- Information valuation skills, including the knowledge of information selection and evaluation, and having skill in information strategies
- Information organizing skills, including having skill in note-taking from books, publications, and saving personal information
- Information interchange skills, including the ability of writing scientific works

The set of these skills is called “Information literacy”. Davarpanah (2007) remarks in this field that those individuals who acquire the information seeking behavior will be capable of searching information independently, and can meet their information needs easily.

### 2.4 Effective Factors on Information Seeking Behavior

Wilson (2000) considers self-efficacy and self-esteem as the most effective factors of information seeking behavior. He believes that a strong feeling of self- esteem for using an information resource leads to wide and accurate use of this resource. Ingwersen (1996) believes that the process of information seeking behavior is associated with cognitive factors such as thinking, perception, memory, recognition, learning, and problem solving. Kuhlthau (1991) emphasizes on the individual aspects of information searchers such as uncertainty, mental confusion, inadequate optimism, relief and satisfaction which can affect searchers’ information seeking behavior.

According to the above discussions, most of the researches reveal that information seeking behavior like any other behavior is the result of a complex interaction. Several inner and outer factors affect the individuals’ information seeking behavior. These factors can be categorized as following:

- Individual factors, including knowledge of information literacynnnn
- System capacities related factors, including user’s interface screen, and methods of indexing and abstractingssss
- Environmental social factors, including demographic factors, social groups, and organization environment
- Information related factors: documentation type, and type and size of the information structure file

### 2.5 Wilson’s Model of Information Seeking

Wilson (2000) applied the process of information seeking as a pattern of problem solving for the first time in 1983. Then, he presented his model of problem solving in 1997 to integrate the researches of this field. In his model of information seeking, searching and using information are related to different processes of problem solving. The processes of problem solving are: identifying the problem, definition of the problem, expression of the problem (if necessary) (Salajaghe, 2005).

Wilson’s model of information seeking includes the following components:

- The context of information need
- Intervening variables
- Activating mechanism
- Information processing and use

In fact, these components are the same constituent elements of information behavior. This pattern shows the cycle of information activities from the process of need creation to the process of using information, and includes several intervening variables which have a noticeable influence on information behavior.

Now we are going to explain every one of the existing components and its related elements.

*The context:* Wilson mentions three human needs on which psychologists are emphasized.

- Physiologic needs, such as need for food, water, shelter, and etc.
- Affective needs, such as need for excellence, mastery, and etc.
- Cognitive needs, such as planning, acquiring a skill, and et

This categorization shows that individual needs are internal and related to each other, and each of them lead to the creation of other needs (Eyni, 2005).

*Intervening variables:* Wilson has mentioned all the intervening factors and depicted them in this model. Mental variables may affect the information seeking behavior as an intervening variable, as well as other intervening variables (e.g., the variables of demographic, environmental, dependent or interpersonal role) (Adhami, 2004).

Individual’s point of view on life and system of value, political orientation, knowledge, style of leaning, emotional variables, individual’s attitude towards innovation, stereotypes, preferences, prejudices, self-perception(self-evaluation of knowledge and skills), interests, knowledge of subject, duty, and information or search system can be mentioned as some psychological variables. Demographic variables include gender, age, social and economic position, education level, job experience, and so on. In this model, psychological and demographic variables have been separated from each other (Ibid.)

*Activating information seeking behavior:* Wilson inserts a concept of activating mechanism between person-in-context and the decision to seek information. He mentions well that every information need offers an incentive to take part in activities leading to seeking information. He looks for an answer in psychology, and other sciences to find out the stimulating and motivating factors of the seeking information. According to him, one of the activating mechanisms can be explained by a stress or coping theory. According to this theory, all the information needs do not make the individual seek information. For example, if an individual is convinced that his possessed knowledge is sufficient to understand the situation and make a decision, he will not engage in seeking information. If he lacks such conviction, this will cause the stress connected with making a mistake, trespassing social or legal norms, financial responsibility or not satisfying others’ expectations. The greater the stress the greater is the motivation to seek information, up to a certain extent where the stress disables these activities. Another activating factor is a necessity to cope with a situation or problem solving. Tendency towards getting a reward includes this feeling of necessity, even if this reward only leads to bringing comfort due to eliminating the feeling of uncertainty (Niedzwiedzka, 2003).

*Information processing and use:* Information gained by a user is processed, becomes a part of his knowledge, and is used directly or indirectly to have an impact on the environment, and as a consequence, create new information needs. Mental and physical information activities create a cycle, in which essential elements of the context determine the individual’s behavior at all stages. Then, gained information becomes a new element in a dynamic system (Wilson, 2004). The totality of human behavior formed in any way (passive or active) leads to the information process and use which are the main objective of the research, But these process and use may change according to the context of information need. It means that information process and use may differ based on the various fields and the context of information need (Gazni, 2002). Wilson’s model provides a useful framework for thinking about the process of data collection in the field of research.

### 2.6 Literature Review

Studying human’s information seeking behavior begun through performing researches by reading and through library. Study on the use of library dates back to 1916s, 1920s and 1930s. Increase in the amount of scientific works after World War II led to holding a conference titled “Royal Society Scientific Information” in 1948 which was a starting point for developing a new approach to the study of human information seeking behavior. Ten years later, this conference was followed in Washington, and some papers were published in the fields of information seeking behavior, information need, and the use of information. Most of the researches undertaken between 1948 and 1965 were related to information need. One of the most rigorous researches in this field is carried out by Varis (1973) in Baltimore, U.S. on citizen’s information needs. In 1980s, researches focused on the study of information need and behavior. In 1981, Wilson stated that information need is not one of the humans’ basic needs such as food and housing but it is a secondary need which results in feel satisfaction with basic needs. Palmer in 1991 showed that the information behavior of scientist can be divided into five groups of information searchers who had different personalities. These five groups were:

- *Non-seekers*, they were not motivated for information seeking
- *Lone, wide rangers*, they liked working alone, often solved their problems by chance, and sought information very widely
- *Unsettled, self-conscious seekers*, they were some fresh researchers, visited the library more frequently than the other groups, consulted their colleagues due to uncertainty about their role and information seeking, and had not chosen the subject of their researches themselves
- *Confident collectors*, they did not exert effort to conscious information seeking but they tried to get new information.
- *Hunters*, they worked in biochemistry. They had their own strategies to cope with the information flow. They used library, and had contact with colleagues both in their own country and abroad. (Palmer, 1991)

Ellis (1989), Ellis et al. (1993), and Ellis & Haugan (1997) studied the information seeking behavior of social scientists, research physicists and chemists, and engineers and research scientists in an industrial firm. Finally, they proposed a model which can be used by the researchers of different fields with a little change. This model is based on the information searching on web. Kuhlthau (1994) examined a model of information seeking behavior which was initially based on a study of high school students. The stages of his proposed model were initialization, selection, exploration, formulation, collection and presentation. According to him, each stage is associated with certain feelings and specific activities.

Mehta and Young (1995) studied the scientists’ scientific and technical information needs. The results showed that 83% of respondents consider the use of the resources and references of the existing papers as one of the most important information resource.49% and 42 % of respondents respectively use databases and light impact discs. 71% of faculty members of the studied university use electronic post to communicate. Getting information of their friends and co-workers was reported as the main reason. Ucak and Kurbanoglu (1998) examined the information need and information seeking behavior of professors in universities of turkey in the fields of engineering, human, and social sciences. The results showed that the researchers’ information need and information seeking behavior depend on their field of research activity, and vary from field to field. Fidel and Efthimiadis (1999) conducted a research about information seeking behavior of engineers. Their result showed that most of the engineers had problems about the way of communication, and were not able to make accurate decision. From among all of the search methods, engineers prefer keyword search method as the most important criteria. Makri et al (2008) investigated the lawyers’ information seeking behavior and presented a model using Ellison’s model. He analyzed several models of users’ information seeking behavior which were at a high level of making abstract. He concluded that Ellis is a potential model among other models.

Chung (2006) performed a research on the information seeking behavior of those individual whose first language is not English. These individuals’ behaviors were studied on a non-English web. He developed a Spanish business Web portal that supports searching, browsing, summarization, categorization, and visualization of Spanish business Web pages. He found that, compared with a Spanish search engine and a Spanish Web directory, it achieved significantly better user ratings on information quality, cross-regional search capability, system performance attributes, and overall satisfaction. Subjects’ verbal comments strongly favored the search and browse functionality and user interface of our portal. As the Web becomes more international, his research made three contributions: (1) an empirical evaluation of the performance level of a Spanish search portal; (2) an examination of the information quality, cross-regional search capability and usability of search engines for the non-English Web; and (3) a better understanding of non-English Web searching. Al-Salem (1989) studied the relationship between different types of education systems and information seeking behavior types among faculty members of the University of Wisconsin-Madison. The results showed that education plays an important role in information seeking behavior. Olaisen (1984) examined the information seeking behavior among faculty members in decentralized systems of Norwegian Universities. The results revealed periodicals were one of the main sources used by the research population. Francis (2005) studied the information seeking behavior among the faculty members of University of the West Indies, St. Augustine Campus in Trinidad and Tobago. She concluded that textbooks and conference papers were the most important preferred resources, and that faculty members prefer electronic resources to the printed resources.

### 2.7 Methodology

The present research is an applied research which surveys the information seeking behavior of Faculty members. Research population consisted of 130 faculty members. Using krejcie & Morgan table, sample size of 97 was obtained. Data collection tool was an information-seeking behavior inventory which had 24 questions based on 5-point likert scale whose validity and reliability has been confirmed using Construct validity and Cronbach’s alpha. After collecting data, they were analyzed in SPSS software. Descriptive and inferential statistics and results will be presented in next section. Our research hypotheses are as following:

H1. There is a significant relationship between English language proficiency, and the use of internet among the faculty members of PNU.

H2. There is a significant relationship between academic rank and work experience of the faculty members and their internet-based information seeking behavior.

## 3. Results and Discussion

### 3.1 Descriptive Statistics

In this section we present descriptive statistics of research variables. Table 1 shows demographic characteristics of the study sample. Table 2 shows the frequencies related to research questions, and Tables 3-5 present descriptive of variables “English language proficiency”, “academic rank”, and “work experience”.

**Table 1:** Demographic characteristics

Measure	N	%
**Age**		
32-47	97	100
Total	97	100

**Academic degree**		
PhD	45	46.4
M.Sc	52	53.6
Total	97	100

**Academic rank**		
Lecturer	45	46.4
Assistant Professor	45	46.4
Associate Professor	4	4.1
Professor	3	3.1
Total	97	100

**Sex group**		
Male	77	79.4
Female	20	20.6
Total	97	100

**Work experience (year)**		
2-13	97	8.2
Total	97	100

**Table 2:** Frequencies of research questions

Questions	Components	Frequency	Percent
*The aim and motivation of seeking information*	Publishing scientific paper	50	51.5
	Presentation at seminars and conferences	36	37.1
	Increasing efficiency in teaching	4	4.1
	Identifying technical sources	4	4.1
	Interest in updating technical information	3	3.1

*How identify information resources*	Referring to bibliography at the end of printed resources	14	14.4
	Using references in electronic resources	3	3.1
	Interviewing and consulting with friends	17	17.5
	Bibliographies	3	3.1
	Abstracts and indexes	4	4.1
	Using internet search engines	56	57.7
	Attending in seminars and conferences	0	0

*Problems in accessing information*	Low-speed internet and network traffic	31	32
	Lack of sufficient proficiency to English language	22	22.7
	Insufficient knowledge of search methods	9	9.3
	insufficient computer skills	14	14.4
	Lack of access to the internet	10	10.3
	Lack of enough time	4	4.1
	Lack of need to these resources	4	4.1
	Unfamiliarity with internet service providers	3	3.1

**Table 3:** Descriptive statistics- English language proficiency

	Mean	N	SD	SEM
English language proficiency	4.13	97	0.702	0.071

**Table 4:** Descriptive statistics-Academic rank

	Mean	N	Std. Deviation	Std. Error mean
Lecturer	3.55	45	0.373	0.055
Assistant Professor	3.26	45	0.251	0.037
Associate Professor	3.240	4	0.161	0.080
Professor	3.233	3	0.140	0.081
Total	3.395	97	0.340	0.034

**Table 5:** Descriptive statistics-Work experience

	Mean	N	SD	SEM
Work experience	1.84	97	1.106	0.112

### 3.2 Inferential Statistics-Testing Hypotheses

In this section we present analysis results of testing research hypotheses.

#### 3.2.1 Hypothesis 1: English Language Proficiency Has Significant Effect on Using Internet among Faculty Members of PNU

One-sample t-test is used to examine first research hypothesis. This test determines that whether there is any significant difference between the observed mean and an estimated value or not. In this research since data are collected based on 5-point Likert scale. So, the mean is between 1 and 5. The researcher’ desired criterion is 3. The results are shown in Table 6.

**Table 6:** One-sample t-test of H1

	Test average=3					

	t	df	Mean differences	Significance level	Confidence interval of the mean differences	

					Lower bound	Upper bound
English language proficiency	15.919	96	1.134	0.000	0.99	1.28

According to Table 6, since the t-test value and significance level (p-value= 0.000) is less than 0.05, so this hypothesis can be accepted; Therefore, at a 95% confidence level it can be said that English language proficiency are effective in using internet among faculty members.

#### 3.2.2. Hypothesis 2: Academic Rank and Work Experience of PNU Faculty Members Are Effective in Their Information-Seeking Behaviors

In this section we used Pearson correlation test to examine the relationship between work experience and information-seeking behavior. Table 7 shows the results.

**Table 7:** Correlation test results of work experience and information-seeking behavior

		Information-seeking behavior	Work experience
Information-seeking behavior	Pearson Correlation(R)	1	-0.742
	Sig.(2-tailed)		0.000
	N	97	97
Work experience	Pearson Correlation	-0.742	1
	Sig.(2-tailed)	0.000	
	N	97	97

Based on Table 7, R=-0.742 which is greater than critical value of 0.205 at 95% confidence level, so there is an inverse correlation between two mentioned variables. Also significance level was 0.000; since it is less than 0.05, so there is a significant relationship between work experience and information-seeking behavior of PNU faculty members in Mazandaran Province.

Now to examine relationship between academic rank and information-seeking behavior we use one-way ANOVA. Results are shown in Table 8.

**Table 8:** ANOVA test results- academic rank and information-seeking behavior

	Sum of squares	Df	Mean score	F	Sig.
Between groups	2.090	3	0.697	7.162	0.000
Within groups	9.045	93	0.097		
Total	11.134	96			

Based on above table, sig.=0.000 and F=7.162. Since sig p-value< 0.05, so with a 95% confidence level we can say that there is a significant difference between academic rank and information-seeking behavior of faculty members.

## 4. Conclusion

In this research, we studied the information-seeking behavior of faculty members in Payame Noor University (PNU) of Mazandaran, Iran. According to the conducted survey, it was found that the most important goal of faculty members was “publishing a scientific paper”, and their least important goal was “updating technical information”. The faculty members mostly use internet-based resources to meet their information needs. Accordingly, 57.7% of them identify information resources via internet research engines (Google, Yahoo, and etc.). We also examined the relationship of “English language proficiency”, “academic rank”, and “work experience” of faculty members with their information- seeking behavior. Results confirmed the significant relationship between them at 95% confidence levels.

## References

[1] Adhami A (2004). What Is Information Seeking And Information Seeking Behavior?. Journal of Informing.

[2] Al-Salem M (1989). An Investigation of the Relationship between Academic Role and The Information-Seeking Behaviour of Adult Education Faculty Members.

[3] Bronstein J (2007). The Role Of The Research Phase In Information Seeking Behavior Of Jewish Studies Scholars: A Modification Of Ellis’s Behavioral Characteristics. Information Research.

[4] Cooke A (2001). A Guide to Finding Quality Information on the Internet: Selection and Evaluation Strategies.

[5] Chung W (2006). Studying Information Seeking On The Non-English Web: An Experiment on A Spanish Business Web Portal. International Journal of Human-Computer Studies.

[6] Choo C. W, Deltor B, Turnbull D (2000). Information Seeking And Knowledge Work on The World Wide Web. Dordrecht.

[7] Davarpanah M. R (2002). Seeking Scientific and Research Information In Print And Electronic Sources.

[8] Davarpanah M. R (2007). Scientific Relation: Information Need And Information Seeking Behavior.

[9] Ellis D, Cox D, Hall K (1993). A Comparison of the Information Seeking Patterns of Researchersin the Physical and Social Sciences. Journal of Documentation.

[10] Ellis D, Haugan M (1997). Modelling the Information Seeking Patterns of Engineers and Research Scientists in An Industrial Environment. Journal of Documentation.

[11] Ellis D (1989). A Behavioral Model For Information Retrieval System Design. Journal of Information Science.

[12] Eslami A, Keshavarz H (2006). A Study on Students’ Skill at Searching For Information in Online Electronic Sources. PhD Thesis.

[13] Eyni A (2005). The Concept of Information Need From the Perspective of Pioneer of Library and Informing Sciences. Journal of Sciences And Informing.

[14] Fidel R, Efthimiadis E. N (1999). Web Searching Behavior of Aerospace Engineers.

[15] Francis H (2005). The Information-Seeking Behavior of Social Science Faculty at the University of the West Indies, St. Augustine Campus. Journal of Academic Librarianship.

[16] Gazni A (2002). Interactional Points Of View in Design Systems of Information Retrieval. Journal of Book.

[17] Ingwersen P (1996). Cognitive Perspectives Of Information Retrieval Interaction: Elements of A Cognitive IR Theory. Journal of Documentation.

[18] Krikelas J (1983). Information Seeking Behavior: Patterns and Concepts. Drexel Library Quarterly.

[19] Kuhlthau C. C (1991). Inside The Search Process: Information Seeking From The User’s Perspective. Journal of the American Society for Information Science.

[20] Kuhlthau C. C (1994). Seeking Meaning: A Process Approach To Library and Information Services.

[21] Lancaster F. W (1986). Vocabulary Control for Information Retrieval.

[22] Marchionini G. M (1995). Information Seeking In Electronic Environments.

[23] Mehta U, Young V. E (1995). Use of Electronic Information Resource: A Survey of Science and Engineering Faculty. Science And Technology Libraries.

[24] Makri S, Blandford A, Cox A. L (2008). Investigating The Information-Seeking Behaviour Of Academic Lawyers: From Ellis’s Model To Design. Information Processing and Management: An International Journal.

[25] Meho L. I, Tibbo R. H (2003). Modeling The Information- Seeking Behavior of Social Scientists: Ellis’s Study Revisited. Journal of the American Society For Information Science and Technology.

[26] Niedzwiedzka B (2003). A Proposed Model of Information Behavior. Journal of Information Research.

[27] Nokarizi M, Davarpanah M. R (2006). The Analysis Of Information Seeking Patterns. Journal of Library and Information Science.

[28] Olaisen J. L (1984). Toward A Theory Of Information Seeking Behavior Amongscientists And Scholars.

[29] Palmer J (1991). Scientists and Information: II. Personal Factors in Information Behaviour. Journal of Documentation.

[30] Saracevic T (2002). Introduction to Information Science.

[31] Salajaghe M (2005). The Presentation of Information Seeking Pattern Resulting from Studying the Information Seeking Behavior. Case Study: Faculty Members of Universities of Medical Sciences In Iran PhD Thesis.

[32] Ucak O. N, Kurbanoglu S. S (1998). Information Need and Information Seeking Behavior of Scholars at a Turkish University.

[33] Varis T (1973). International Inventory of Television Program Structure and the Flow of Programs Between Nations.

[34] Wilson T. D (1981). On User Studies and Information Needs. Journal of Documentation.

[35] Wilson T. D (2000). Human Information Behavior. Informing Science.

[36] Wilson T. D (2004). Information Seeking Behavior and the Digital Information World. European Science Editing.

